# Socio-economic baseline for oil-impacted communities in Ogoniland: towards a restoration framework in Niger Delta, Nigeria

**DOI:** 10.1007/s11356-024-32805-0

**Published:** 2024-03-14

**Authors:** Kabari Sam, Nenibarini Zabbey, Ijeoma Favour Vincent-Akpu, Gentle Komi, Peter Oghogho Onyagbodor, Bolaji Bernard Babatunde

**Affiliations:** 1 School of Environment, Geography and Geoscience, University of Portsmouth, Burnaby Road, Portsmouth, PO1 3QL UK; 2 Department of Marine Environment and Pollution Control, Nigeria Maritime University Okerenkoko, Warri, Nigeria; 3 Department of Fisheries, Faculty of Agriculture, University of Port Harcourt, East-West Road, PMB 5323, Choba, Port Harcourt, Rivers State Nigeria; 4 Environment and Conservation Unit, Centre for Environment, Human Rights and Development (CEHRD), D-Line, Port Harcourt, Rivers State Nigeria; 5 Department of Animal and Environmental Biology, Faculty of Biological Sciences, University of Port Harcourt, PMB 5323, Choba, Port Harcourt, Rivers State Nigeria

**Keywords:** Alternative livelihoods, Oil contamination, Artisanal refining, Ramsar, Ecological restoration

## Abstract

This study documents the socio-economic baselines in selected oil-impacted communities prior to the commencement of the Ogoni clean-up and restoration project. Adopting mixed approach consisting of semi-structured interviews, focus group discussions (FGDs), key informant interviews (KIIs), and household surveys, we surveyed the pre-remediation socio-economic conditions in the Ogoniland communities between July 2018 and March 2019. Results indicated that almost all respondents (99.6%) agreed that the smell of petroleum products or crude oil was evident in the air they breathed even as there were visible black particles (soot) in the respondents’ nostrils, on their clothes, and in water. The respondents described the ambient air as smoky and choked with an offensive smell. The household waters were smelly, brownish, or oily, and most respondents (76%) cannot afford to treat their water. Forty-two percent of the respondents who relied on fishing and farming for a living sought for alternative means of subsistence and acknowledged that oil pollution caused stunted growth and low crop yield. The majority of respondents (91%) reported falling fish catches, while the fish caught smell and taste of oil, lowering their market value and posing a potential health risk to consumers. It is evident that oil pollution has impacted the socio-ecological values and sustainable livelihood in Ogoniland. This study provides baseline data for monitoring post-remediation socio-economic improvements in Ogoniland. It also highlights areas of urgent intervention to improve livelihood, and access to basic amenities (e.g., potable drinking water), waste management infrastructure, and statutory policy changes for sustainable development in Ogoniland.

## Introduction

Environmental and socio-economic impacts are commonplace in natural resource-dependent economies. Given the increasing awareness and the need to meet global goals including the United Nations Decade on Ecosystem Restoration (UNDER) and the Sustainable Development Goals (SDGs), environmental remediation and ecosystem restoration is gaining traction in developing countries. In Nigeria, for example, the Niger Delta region is currently hosting the Ogoni remediation and Bodo Creek restoration projects. Both projects would require environmental and socio-economic baselines to measure the progress of remediation and restoration projects.

Natural resource mining is associated with severe environmental, economic, and socio-cultural impacts. Countries, particularly developing ones, faced with these impacts struggle to contribute to global visions, including the Sustainable Development Goals (SDGs), the (UNDER), and several climate-related actions. In developing countries such as Nigeria, Angola, and Equatorial Guinea, host communities have reported high likelihood of exposures to hazards associated (e.g. polyaromatic hydrocarbons, PAHs) (Sam et al. 2023a). Research has also linked irresponsible natural resource mining to severe public health and environmental risks and loss of livelihood that often dislocate the local economy (Lamine and Xiong 2013; Sam and Zabbey 2018; Sam et al. 2024). Within Nigeria, the attendant impacts of oil exploration and exploitation have been attributed to the neglect and lack of duty of care of the oil industry operators (UNEP 2011), weak legislation (Sam et al. 2015), overlapping regulatory responsibilities (Sam et al. 2017a), poor enforcement, and an increasing lack of commitment to sustainable environmental management (Ogunkan 2022). As a result, communities that host natural resource mining activities face severe consequences that limit their access to sustainable traditional livelihoods, potable drinking water, fertile soil for agriculture, and a flourishing ecosystem that can support and improve access to ecosystem goods and services needed for life sustenance (Pegg and Zabbey 2013).

The magnitude of socio-ecological impacts occasioned by the oil industry operations in Nigeria has been attributed to gaps in sectoral legislation. For example, oil mining activities began in Nigeria in 1958 and the first legislation, the Petroleum Act 1969 (which reflected on environmental management), came 10 years later (Sam et al. 2017c), and the country is yet to develop comprehensive legislation for oil spill detection and responses more than 50 years after the full operationalization of the oil and gas sector (Sam et al. 2017c, 2022). As a result, the Niger Delta region, the hub of oil exploration and production in Nigeria, has experienced large-scale oil spills. Consequently, the land, water, fauna, flora, and air in the region are known to be severely contaminated from incessant oil spills (Elum et al. 2016; Sam et al. 2022).

## Socio-economic situation in the Niger Delta and Ogoniland

The situation in the region is characterized by an absence of trust between relevant stakeholders; e.g. host communities do not trust international oil companies (IOCs) (UNEP 2011; Sam et al. 2022), following decades of oil spills and the exposure of community members to plausible risks without remediation or management action (Pegg and Zabbey 2013). In addition, corporate social responsibility projects that could have provided social support structures for the oil-impacted communities have failed in many instances (Sam et al. 2024) because of exclusion and decades of frustration. As a result of this neglect and the inability of local communities to access clean land or alternative livelihoods, the communities are often characterized by protests (UNEP 2011), inter-communal conflicts, and violence as local communities struggle to farm on few available uncontaminated lands (Osuagwu and Olaifa 2018).

The communities in Ogoniland are exposed to petroleum pollutants at elevated concentrations through different pathways including outdoor air, food, drinking water, and dermal contacts with contaminated soil, sediments, and surface water (UNEP 2011). As a result, drinking water sources have been contaminated, farmlands polluted, livelihoods destroyed, local economy dislocated, and cultural values eroded. For example, UNEP in 2011 reported that drinking water sources in Nsisioken (a community in Ogoniland) contain benzene concentrations 900 times greater than the World Health Organization–recommended level (UNEP 2011). The WHO’s global health observatory records an average life expectancy in Nigeria as being less than 55 years in 2020 (WHO 2023); this could be lower in Ogoniland communities, where community members have lived with chronic oil pollution throughout their lives. Local communities have continually expressed displeasure, distrust, and dissatisfaction with the level of environmental degradation and socio-economic losses via protests and agitations.

The results of the Environmental Assessment of Ogoniland undertaken by the United Nations Environment Programme (UNEP) in 2011 provided evidence of extensive soil and groundwater contamination in the area. The UNEP report provided evidence that locals in Ogoni communities were exposed to hydrocarbons through different pathways, including inhalation, ingestion, and dermal contact with polluted soil or water, and that these had cumulatively affected the health of local communities in the area (UNEP 2011). In 2016, the government flagged off the remediation project, with the inauguration of the Hydrocarbon Pollution Remediation Project in 2017; however, it took years for actual remediation work to commence as HYPREP needed to set up governance and administrative structures. In 2019, 8 years after the UNEP assessment report, HYPREP commenced the implementation of the recommendations of the UNEP report. Given the dynamic nature of the Niger Delta, and specifically the Ogoni environment, there is a high probability that the status of many environmental and socio-economic parameters investigated by UNEP might have changed.

A significant challenge for the remediation of polluted sites in Ogoniland is the establishment of baselines for the restoration of certain socio-economic and environmental parameters; e.g. biodiversity restoration will be dependent on establishing baselines for the microbial community. Thus, achieving near-pristine environmental restoration could be challenging as there are no baselines documented prior to the five-decade oil spills. To fill this gap, this study aims to provide a pre-remediation baseline for purposes of monitoring and referencing, along the project lifecycle. Mainly, the study presents the current situation of Ogoni communities regarding oil contamination and provides information and tools to help remediation practitioners and regulatory agencies reach their goals and meet community expectations.

## Governance and regulatory framework for contaminated land management in Nigeria

The Nigerian contaminated land regulatory framework transcends three eras. First, the era of no regulation (1956–1968), the era of non-specific regulation (1969–2001), and the era of near-specific regulation (2002–to date) (Sam et al. 2017c). During the era of no regulation, oil contamination incidents were on the rise, as government focused on profit maximisation (Sam et al. 2017a). This elicited a response from the government and marked the beginning of the period of non-specific legislation.

The development of the Petroleum Act (1969) introduced an overarching law that empowered the Minister of Petroleum Resources to make regulations for the prevention of environmental pollution in the country. In 2002, a first attempt to develop a near-specific contaminated land regulation was initiated with the development of the Environmental Guidelines and Standards for the Petroleum Industry in Nigeria (EGASPIN) 2002. The EGASPIN has been revised a few times with the 2018 version being the most recent; however, the regulation is yet to meet stakeholders’ expectations (Sam et al. 2022; Sam et al. 2023b). For example, the UNEP report of 2011 recommended a review of the EGASPIN with specific attention to the intervention and target values of the regulation (UNEP 2011); however, this is yet to be revised (Sam et al. 2022).

**Fig. 1:**
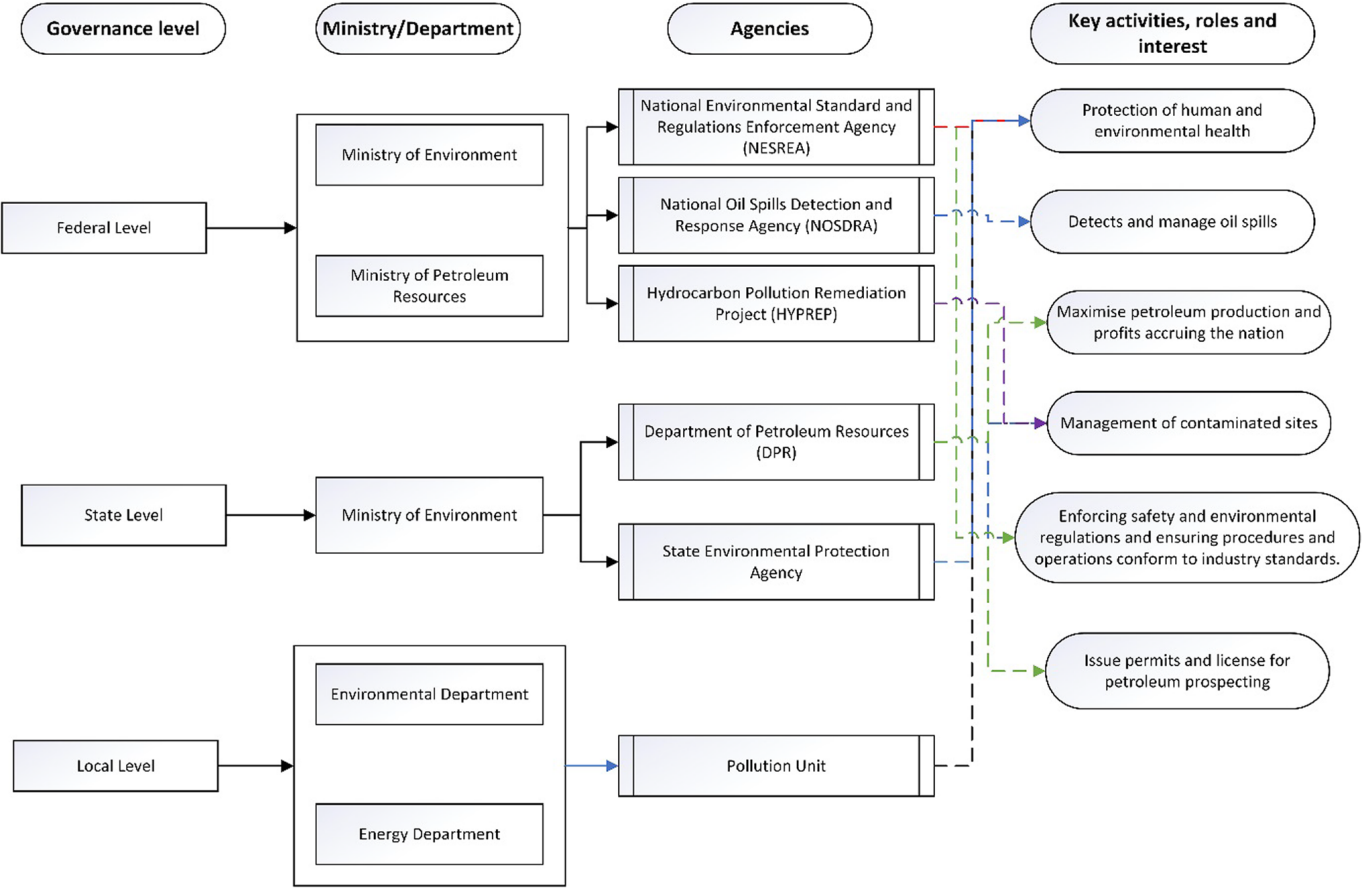
Governance complexities and overlapping roles of environmental protection agencies, departments, and units across the three tiers of government in Nigeria. Functions performed by each agency are represented in colour: NESREA (red), DPR (green), NOSDRA (orange), HYPREP (purple), State Environmental Protection Agency (blue), Pollution Unit (black) (modified from Sam et al. 2017c)

A more pertinent challenge of the current contaminated land regulation regime is the conflict of interest between regulatory agencies. Different states of the Nigerian federation have their independent environmental protection agencies, whose functions often overlap with the federal agencies (Fig. 1; Sam et al. 2022). The activities of these institutions are interrelated and overlap in many areas, which has led to unnecessary prolonged bureaucracies, duplications, and inefficiency (Sam et al. 2022). In addition to the governance problems of contaminated land management in Nigeria (Rim-Rukeh 2015; Ambituuni et al. 2014; Sam et al. 2015), the regulatory thresholds or permissible levels used to make judgments on whether or not a land is contaminated are weak and inadequate to govern a sustainable remediation process (UNEP 2011; Sam et al. 2022).

The current contaminated land management regulation (EGASPIN) was developed by the defunct Department of Petroleum Resources (DPR) but largely implemented by another agency—the National Oil Spills Detection and Response Agency (NOSDRA). Although this has been extensively addressed in Sam et al. (2017c), NOSDRA needs to ratify bespoke thresholds adopted by ongoing remediation projects in the Niger Delta including Ogoniland. For example, HYPREP is reported to adopt a target value of 1000 mg/kg for total petroleum hydrocarbon in soil, against the 50 mg/kg stipulated in the regulation (Sam et al. 2022). There are concerns that the adopted target value is higher than the threshold in the regulation against the recommendation of UNEP (Sam et al. 2022), even as different risk assessment methodologies adopted by remediation practitioners introduce substantial uncertainty in the final risk assessment decisions (Sam 2023). These factors dissuade the trust and confidence of stakeholders in the process. Thus, a ratification of bespoke thresholds by the regulatory agency might restore confidence in the bespoke threshold.

These overlapping functions, complex governance structures, and ineffective regulatory thresholds and frameworks have been reported to impede sustainable management of contaminated land elsewhere (Bardos et al. 2011, 2016; Hou et al. 2014; Hou and Al-Tabbaa 2014; Lee et al. 2023). Therefore, achieving sustainable management of contaminated sites in the Niger Delta region will require addressing these issues and, thus, constitute parameters to be monitored for measuring sustainable contaminated land remediation and restoration.

## Materials and methods

### Study area

**Fig. 2:**
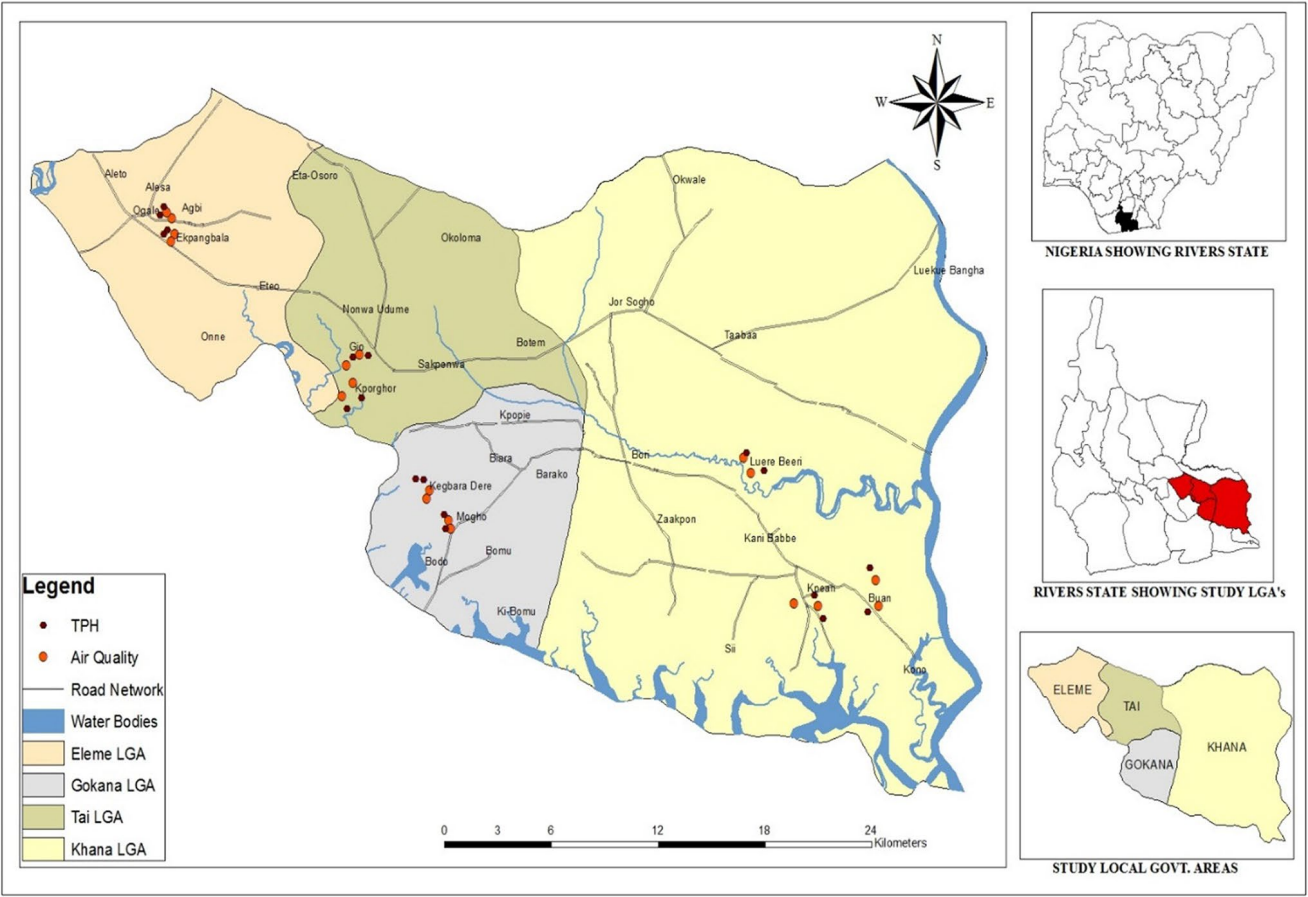
Study area showing sampled communities

Ogoniland is situated in Rivers State, Nigeria, and covers around 1000 km2. The population is approximately 831,726 persons, who are predominantly fisherfolks and farmers, residing in 226 communities in four local government areas (LGAs): Eleme, Gokana, Khana, and Tai (Fig. 2). The local population are predominantly farmers and fisherfolks that depend on clean land and rivers as livelihood structures (Sam and Zabbey 2018). With increased land and water contamination, there is a remarkable decrease in dependence on these resources which has resulted in reduced income earnings in local communities (Nwozor 2020).

### Research design

The study used a mixed approach mainly qualitative to elicit socio-demographic data, status of the environment, and waste management practices from the study population. These were validated with the quantitative approach using structured questionnaire administered to 269 adults in household survey. The total population for the four Ogoni local government areas is given as Eleme LGA, 190,884; Tai LGA, 117,797; Khana LGA, 294,217; and Gokana LGA, 228,828. This made for a total Ogoni population of 831,726 (UNEP 2011). The study applied the Yamane formula for determining sample size (Chaokromthong and Sintao 2021; Louangrath 2017) for a finite population using the population value of 831,726. This enabled the researchers to arrive at a sample size of 399.9. However, given the limited time for the data collection phase of the study, 269 responses were retrieved from respondents (Sam and Zibima 2023).

### Sampling procedure and data collection

Nine communities were sampled (Table 1) for this research, covering oil-impacted and non-oil-impacted communities in Ogoniland. Eight oil-impacted communities and one control community were sampled. However, during the study, it was discovered that the control community may have experienced secondary impact in the past through influx from oil-impacted communities. This is a common experience in Ogoni communities and other communities across the Niger Delta (Albert et al. 2018). An “oil-impacted community” is described as a community that has either experienced oil spills and is to be remediated or a community that has oil pipelines criss-crossing it. A “non-oil impacted community” is one that has neither experienced oil spill nor oil infrastructure such as pipeline. The non-oil-impacted community served as control for the research.

**Table 1:** Communities and local government areas sampled

	Community	LGA	Status
1	Agbi	Eleme	Impacted
2	Ekpangbala		Impacted
3	Gio	Tai	Impacted
4	Kporghor		Impacted
5	Kegbara Dere	Gokana	Impacted
6	Mogho		Impacted
7	Kpean	Khana	Impacted
8	Buan		Impacted
9	Luere Beeri		Not impacted

### Stakeholders’ engagement

Stakeholders, including non-governmental organisations, academia, lawmakers, policymakers, community leaders, regulators, the armed forces, and the diplomatic community, were identified and mapped from previously conducted research in the literature (e.g. the UNEP report) and the scoping mission that led to this study. Identified stakeholders were contacted via phone calls and emails (Prpich et al. 2019; Sam et al. 2017c). Other stakeholders for the engagement were identified through the snowball approach (i.e. respondents in this research identified other stakeholders that participated in the research). Stakeholders (Table 2) from 14 key organizations participated in the various data collection engagements conducted in November and December 2018. Stakeholder engagement activities, including interviews and workshops, were organised in the nine communities (Table 2), and in Port Harcourt, and Abuja.

**Table 2:** List of stakeholders engaged during KII

Organisation	Number of delegates	Location
Facility for Oil Sector Transparency and Reform in Nigeria (FOSTER)	2	Abuja
Cordaid Nigeria	2	Abuja
Chair of mediation process for Bodo Community	1	Abuja
Federal Ministry of Health, Div. of Environmental Health	1	Abuja
National Oil Spill Detection and Response Agency (NOSDRA)	2	Abuja
Federal Ministry of Environment, Div. of Environmental Impact Assessment	1	Abuja
Member of National Assembly, Federal Constituency	1	Abuja
Amnesty International	1	Abuja
Netherlands Embassy in Nigeria	1	Abuja
Nigeria Security and Civil Defence	2	Eleme
Hydrocarbon Pollution Remediation Project (HYPREP)	2	Port Harcourt
Stakeholder Democracy Network Nigeria (SDN)	2	Port Harcourt
National Oil Spill Detection and Response Agency (NOSDRA), Rivers State	2	Port Harcourt
Joint Task Force (JTF)	2	Gokana

### Focus group discussions (FGDs)

**Fig. 3:**
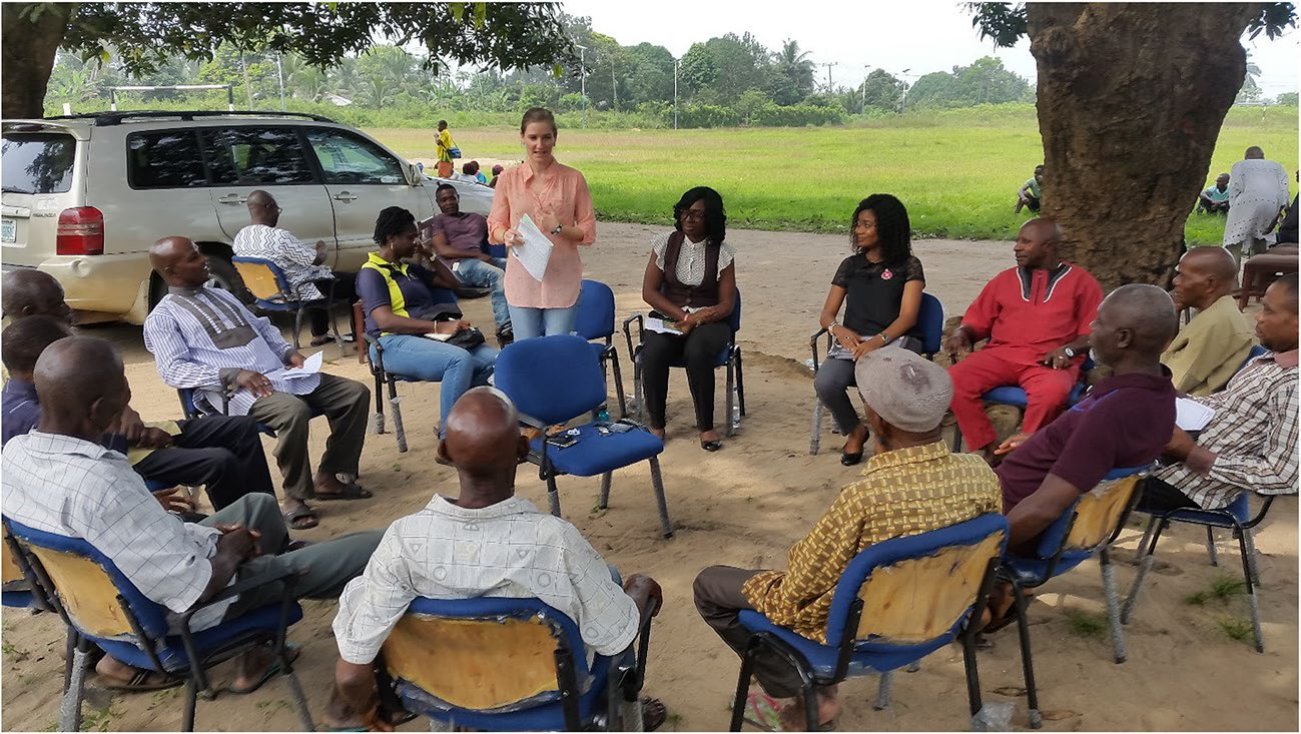
FGD with chiefs in session

**Fig. 4:**
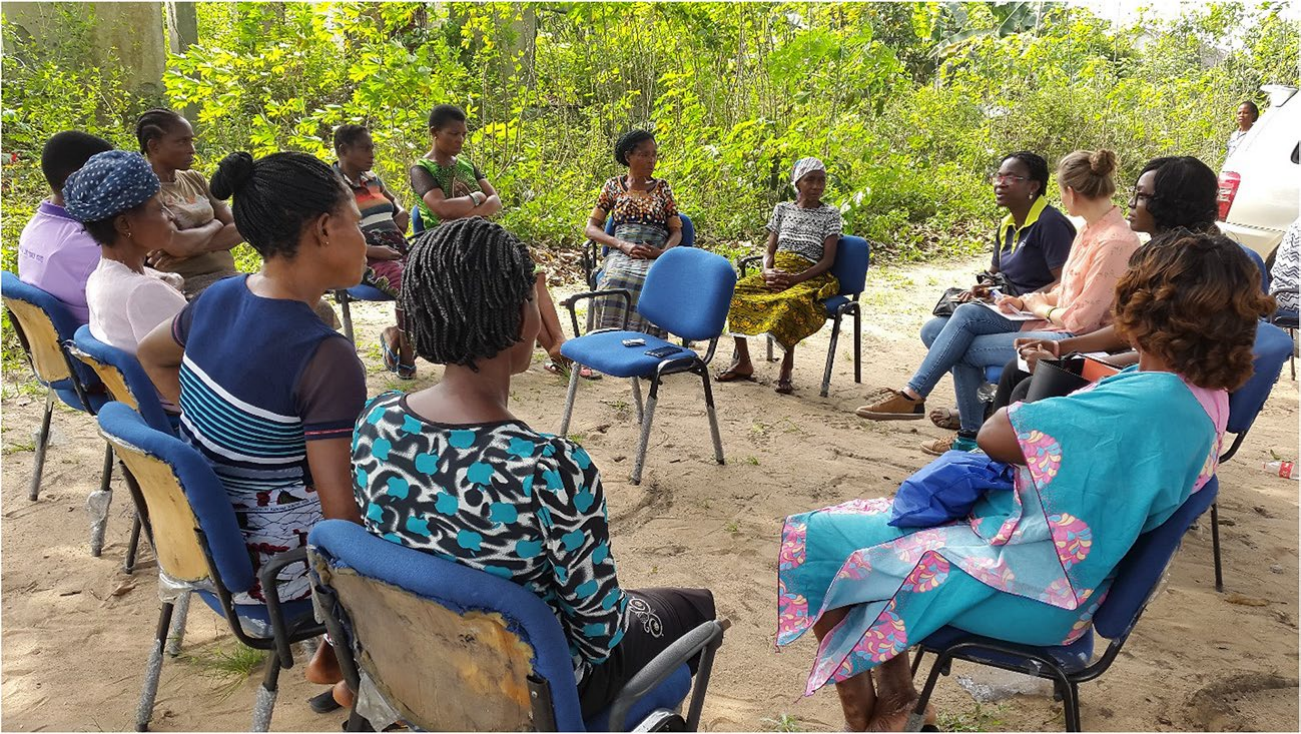
FGD with women group in Kporghor community

**Fig. 5:**
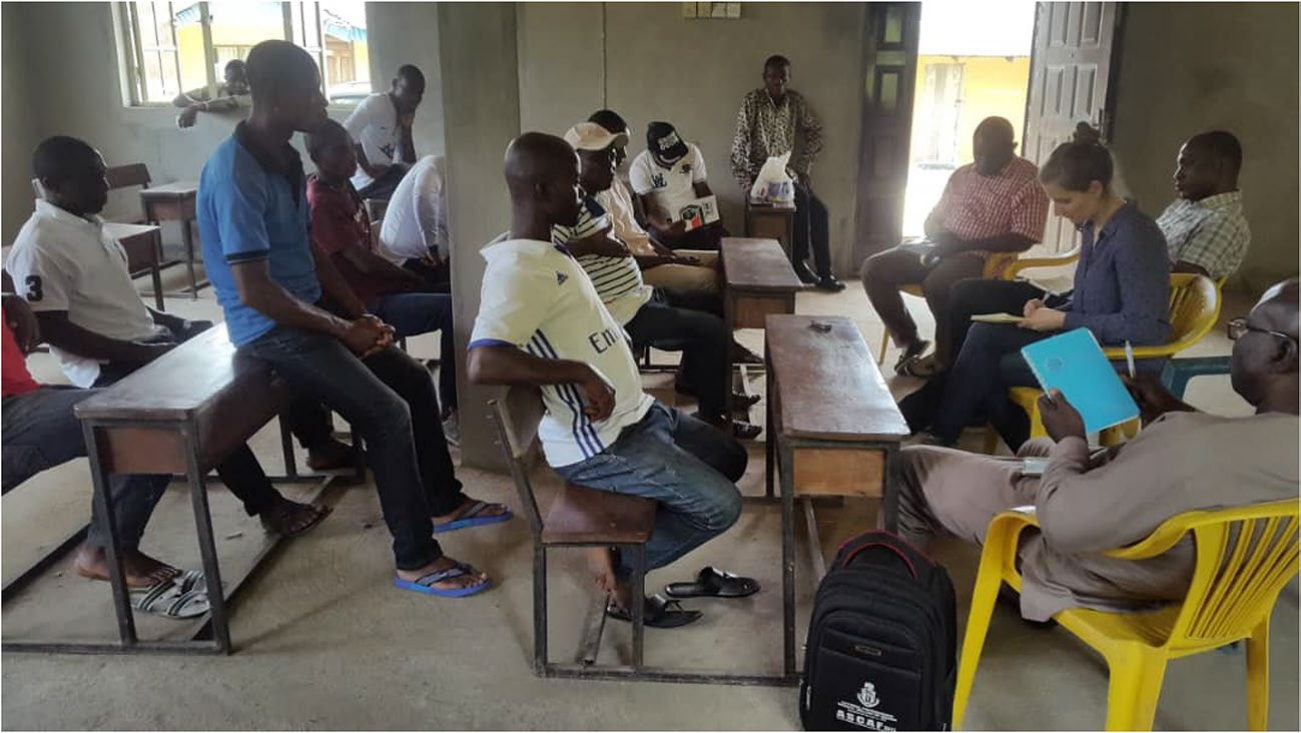
FGD with the youths in one of the communities

In each community, three FGDs were held for the Council of Chiefs, women, and youths, respectively (Figs. 3, 4, and 5), between July 2018 and March 2019. In all, 27 FGDs were held across the different locations. This strategy provided a participatory platform for all stakeholders to express themselves without fear and anxiety. When possible, the first FGD was held with the Council of Chiefs, the traditional rulers of the community, and this gave the team the opportunity to ask their permission to engage with other groups—which is a cultural requirement.

The conversation was semi-structured, and the participants were invited to discuss issues in depth and ask follow-up questions. Women were provided with a safe space to address sensitive issues, which created an opportunity for them to be open as the discussion was guided and prompted by females. The youths were also separately engaged to foster trust and confidence while they expressed their viewpoints on the issues raised during the interview.

### Household survey (questionnaire)

Household surveys were conducted with structured questionnaires, which were administered to 30 randomly selected households in each of the nine sampled communities (270 households were sampled across nine communities). The questionnaire targeted key issues in each field to obtain a broad scope of answers from different participants.

### Data analysis

Data from the household surveys were compiled, cross-checked for accuracy, and entered into Microsoft Excel 2010 on Windows spreadsheet. The datasets were then analysed in the SPSS Statistical Software package, version 22, and presented in tables and charts. Frequencies and basic descriptive statistics were calculated, including cross-tabulations to determine potential relationships between key variables. Audio recordings of participants’ submissions during the FGDs were transcribed verbatim as narratives. The transcripts were coded and analysed using thematic content analysis based on the study objectives.

### Quality control

The semi-structured and structured questionnaires were written and presented to social science experts at the University of Port Harcourt, Rivers State, The Centre for Environment, Human Right and Development (CEHRD), Port Harcourt, and the LKL International Consulting Inc. Montreal, Canada, for content validity. The extent to which the items in the instrument represent the content of the attribute being measured (Amin 2004). To ensure reliability (accuracy and consistency), the instruments were pretested on a different population before being taken to the field.

## Results and discussions

### Social demographic characteristics of survey respondents

The study included 269 adult residents from nine communities, ranging in age from 20 to 80 years old. They comprised 137 (52.7%) males and 123 (47.3%) females. The occupations of the participants ranged widely from farming, fishing, teaching, and local government workers to artisans (apprentices/trainees in skills), trading, and studying. The majority of respondents (47%) had a high school education, while 28.1% had tertiary education (Table 3).

**Table 3:** Respondents’ demographic characteristic

Variable	Number of respondents	Percentage (%)
*Age, n* = *269*		
≤ 30	65	24.7
31–50	136	51.7
51–70	54	20.5
≥ 71	8	3.1
*Gender, n* = *260*		
Male	137	52.7
Female	123	47.3
*Education, n* = *249*		
Primary	62	24.9
Secondary	117	47.0
Tertiary	70	28.1
*Occupation, n* = *252*		
Farmers	112	44.4
Fisherfolks	19	7.5
Traders	42	16.7
Civil servants	42	16.7
Artisans	31	12.3
Students	6	2.4

### Community perception on contamination

#### Contamination of air

In the sampled communities, the smell of crude oil and petroleum products was a common experience. From the FGDs, it was gathered that there is constant soot (black carbon) in the air, most likely occasioned by the artisanal refining of crude oil. “Our air is much polluted, and this is affecting our health: you cannot leave your clothes on the line overnight, because they will be stained with black soot”, a community member commented. Similarly, another youth group emphasized that their white clothes get stained with soot when exposed to air, which was echoed by different community groups. Overall, 99.6% of the surveyed respondents ticked “yes”, that their air was contaminated. This corresponds to a recent research conducted in impacted communities in the Rivers State, which revealed exposure to high levels of soot in the air (Whyte et al. 2020; Zabbey et al. 2021). The respondents described their experiences as including having black particles or stains in their nostrils, especially in the morning; smoky air, choking or offensive smells in the air, stain on clothes; and discolouration of water.

#### Contamination of water resources

Rivers, streams, and creeks transverse the entire Ogoniland region with their daily activities and cultures deeply connected to their natural water sources (Eriegha and Sam 2020; Zeeuw et al. 2018). The water bodies serve as sources of water for drinking, cooking, laundry, bathing, and other domestic activities. In addition, the water bodies form a huge part of their livelihood for fishing (Sam and Zabbey 2018). The majority of participants at the FGDs perceive their water bodies to be contaminated (Table 4). While most of the communities depend solely on the contaminated water, some communities have found alternative sources of drinking water (Sam et al. 2022). Over 76% of the respondents in the communities do not treat their water before using it because they cannot afford to do so. Respondents at FGDs reported that community members buy water for domestic use, even when they cannot attest to the quality or source of the water. The communities used their streams for washing, drinking, and other cultural activities before they were degraded by oil spills. Some sites for clean-up are connected to the source of drinking water implying that the oil from the polluted site flows into the drinking water source, making it unsuitable and unusable. The alternative source of water is boreholes (provided by few private individuals in the community), which, according to the elders, are not safe for use since they originate from the same polluted aquifer. A community member asserted that “contamination of their environment has increased”. For instance, when water from the borehole is kept for 2 days, it changes colour, produces a taste, and stains the container.

**Table 4:** Stakeholders’ perception of water quality in Ogoniland

Variable	Frequency	Percentage%
Contamination of water		
High	224	84.53
Medium	30	11.32
Low	11	4.15
Total	265	100
Contaminated waters		
Stream	218	81.04
Pond	83	30.86
Well	177	65.80
Borehole	145	53.90
Characteristics of household water		
Brownish	120	44.61
Oily	177	65.80
Smelly	175	65.06
Has crude oil	130	48.33
Treatment of drinking water		
Yes	64	23.79
No	205	76.21
Total	269	100

^*^Responses from household survey

Community leaders are primarily concerned about the palliative measures promised by the Federal Government before the clean-up which include potable water and electricity, amongst others. Although the level of water pollution is not uniform across Ogoniland, some communities are severely impacted while others are slightly impacted. The contaminated river has in turn affected their aquatic ecosystem and resources such as fish, periwinkle, and crabs. Oil film or black mud is seen in/on their fishes which reduces the market value, is less attractive to buyers, and is toxic when consumed.

Similarly, the youths and chiefs in the communities expressed concerns that “the borehole water was not safe for drinking because of black particles and oil film in it, and the government should come to their rescue”.

Agbi community in Eleme LGA has no stream or river. The sources of water are dug wells and boreholes at a few houses. The water from the borehole had black suspended particles when collected in buckets, as it was neither filtered nor treated. In order to meet their daily water needs, communities buy water from water vendors (commonly called “aboki”) who bring it in from outside the community. A community member asserted thus: “We spend over N500, the equivalent of USD $1, daily to buy water”.

Similar situations exist in the Ekpangbala community in Eleme LGA. Due to the level of contamination (Fig. 7), evidenced by the oil sheen on waters derived from boreholes and wells, water is bought from water tankers instead of the traditional sources (river, stream, well, and borehole), as was the practice prior to contamination. While the source of these waters sold by vendors cannot be ascertained, the community had no alternative but to patronise such services. A respondent from the community stated that “all our water sources are contaminated, both ground and surface water, we buy water from water tankers or go to nearby communities to fetch it”. Given that most community members might not have the resources to patronise water vendors, they resort to using the polluted water that is available. This, as expected, could potentially result in severe health implications such as skin rash, respiratory disease, and blood poisoning (Hasan et al. 2019; Ite et al. 2018; Li and Wu 2019).

Communities in Khana LGA, such as Kpean, has quite a number of surface water bodies. The major river is “Mui-teegu”, with its tributaries, which are impacted by oil, as stated by the different groups and confirmed by data from the sample analysis. Luere Beeri community (the control) has three streams: “Maabekpabee”, “Maanyonaie”, and “Maayorgbara”. During the FGDs, the community believed strongly that the streams were impacted regardless of the absence of oil spill record. They believed that oil spills from neighbouring communities may have compromised the quality of their surface water because of interconnectivity (Fig. 8). Although the community feel that their solar-powered borehole in the community primary school is fit for drinking, they mentioned that they also depend on rainwater in addition to the solar-powered borehole.

The situation is not different in impacted communities in Gokana LGA. The elders said that Kegbara Dere has three boreholes that always have an oil sheen on them, and even on run-offs during rainfall. The youths confirmed that they use borehole water for other activities but buy drinking water. In Kegbara Dere, the “Op-dee” stream used to be the only source of drinking water before the oil contamination, and the other two rivers where they fish are highly impacted. In addition, they complained that they could smell crude oil in the air (which was obvious during data collection in the community), their surface water is dirty, and the periwinkle and fish were bitter, indicating contamination.

Tai LGA’s communities drew drinking water primarily from streams and wells. A community member in Gio complained: “We only use the well water, because the stream has oil floating on it, even our wells smell of petroleum product”.

**Fig. 6:**
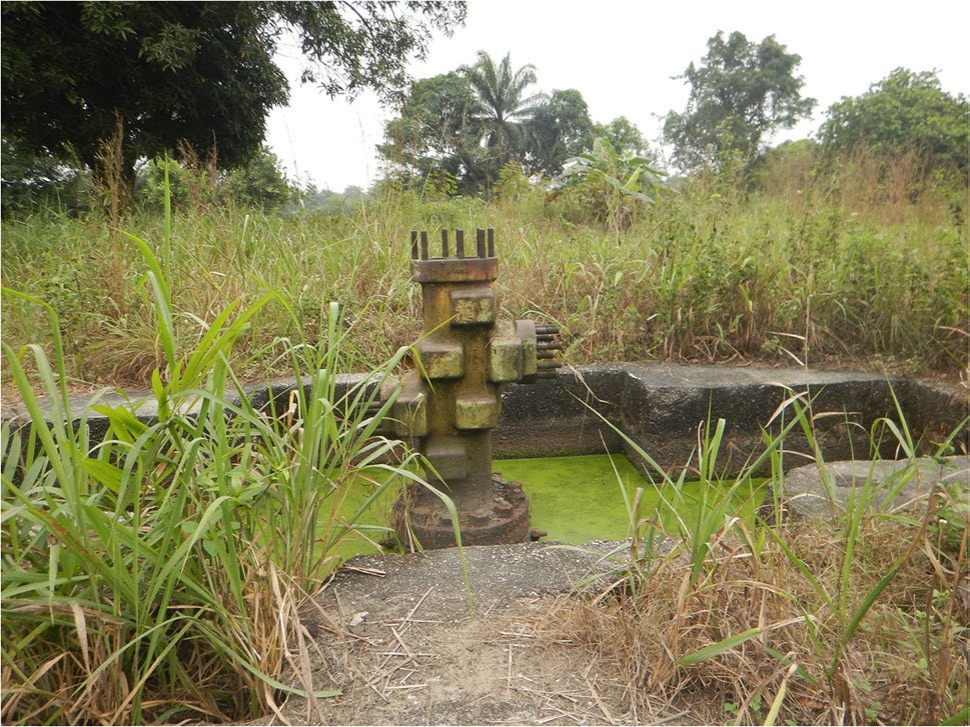
A well-head near residential area in Gokana local government council

Access to safe and potable drinking water, whether from streams, rivers, wells, or boreholes, is at critical levels in these communities, as the surface and ground waters are contaminated with crude oil and not fit for consumption. Oil well heads (Fig. 6) commonly seen in many Ogoni communities are a reminder of the cause of pollutions and environmental degradations in Ogoniland.

**Fig. 7:**
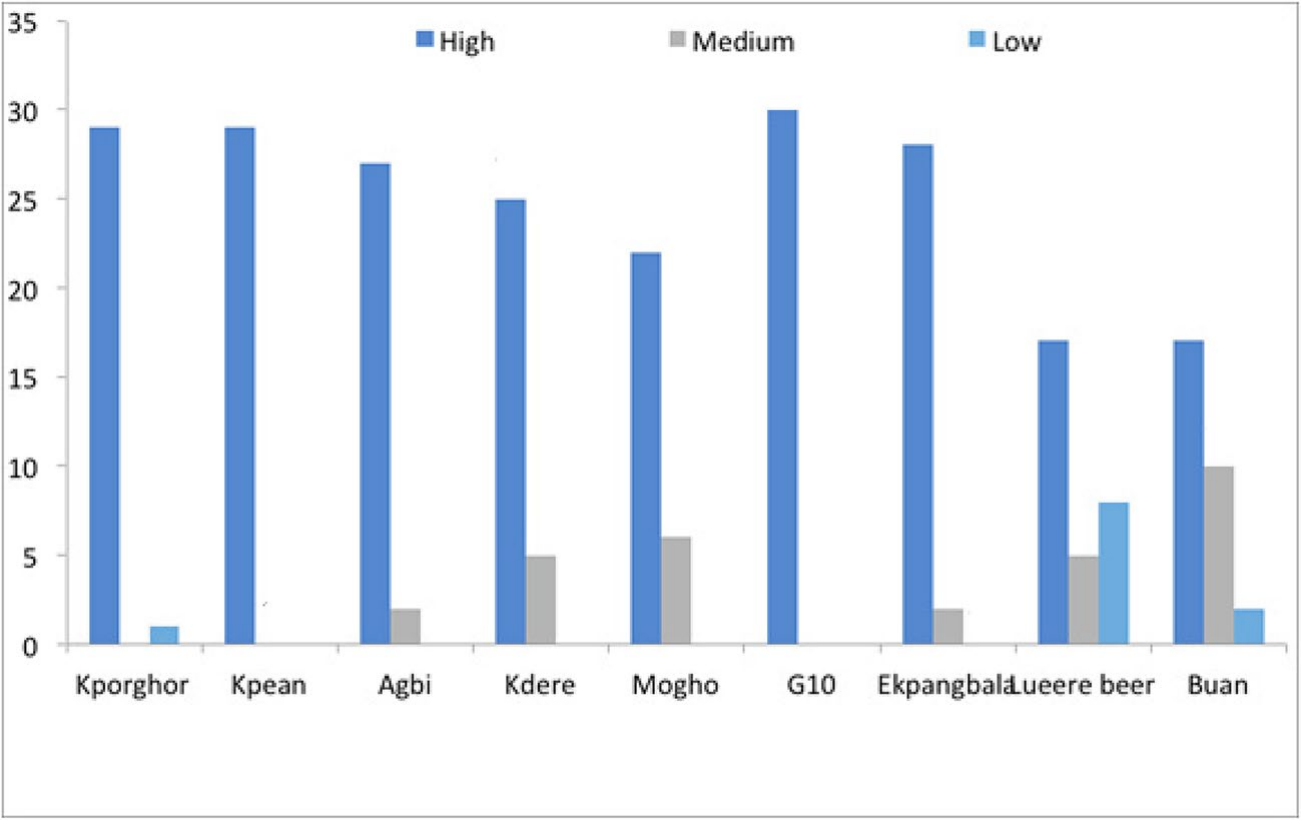
Levels of contamination from household survey

Over 85% in the household survey rated water contamination as high (Fig. 7). The degree of contamination of the water sources, as indicated by the respondents, varied (Table 4).

**Fig. 8:**
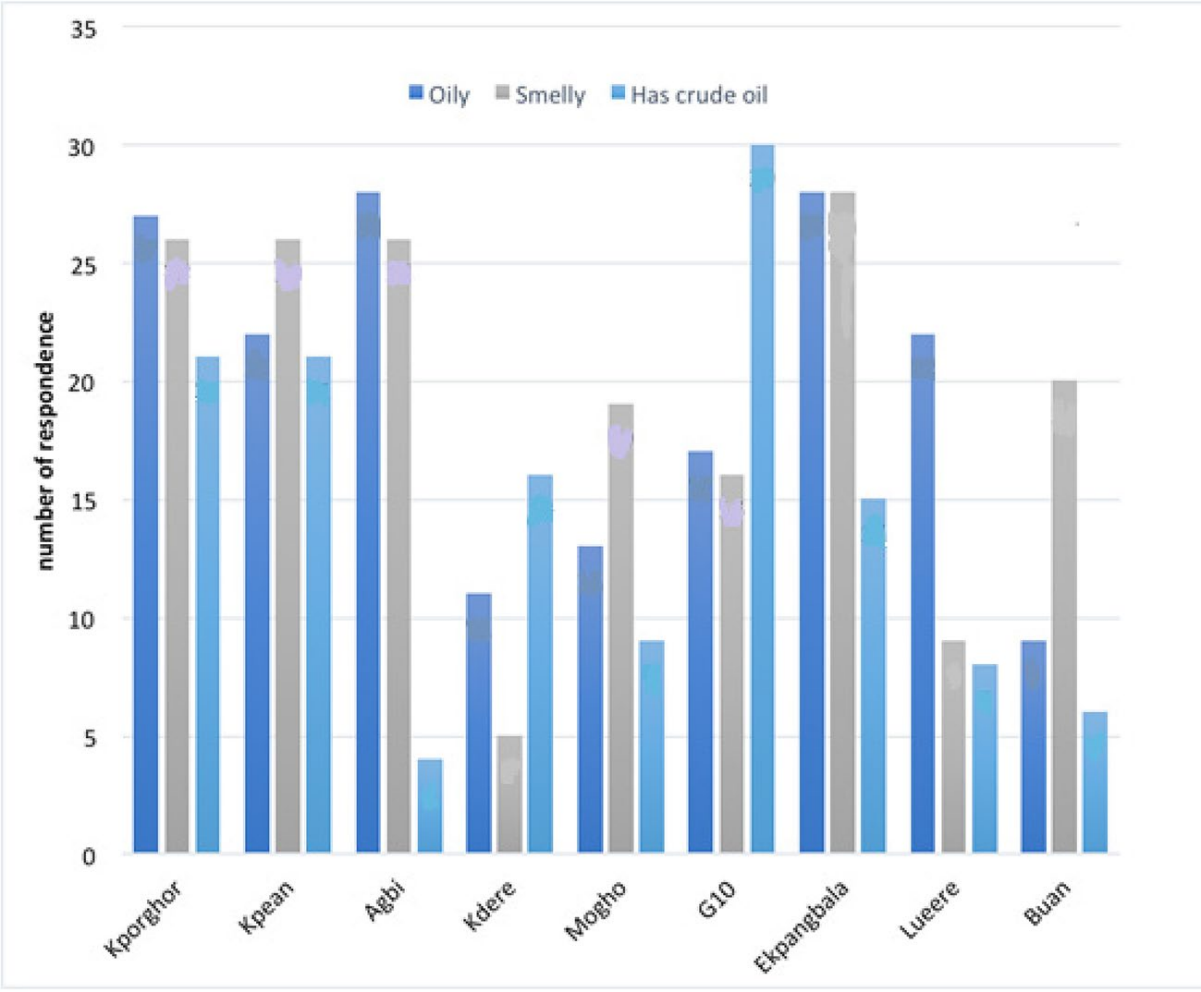
Respondents’ characterization of available water in communities

Common characteristics of household water indicated by stakeholders from each community are shown in Fig. 8. Despite the level of contamination, 76.21% of the villagers do not treat the water before use, but 23.79% do so by boiling it (Table 4).

#### Contamination of land and crops

Ogoniland has alluvial and arable land suitable for variety of agricultural ventures. The people of Ogoni used to be predominately farmers and fisherfolk, though some engaged in trading, crafts, and civil service (Fentiman and Zabbey 2015). This has changed over a five-decade period, following multiple oil spills that have polluted the land. As a result, land use across impacted communities has changed.

For instance, a community member in Agbi explained that ‘‘the land in the area was once used for cultivation of cassava and cocoyam, but now it can no longer be cultivated’’. Another respondent said, “The area, before the havoc done by the oil spill, was the sacred land of the Agbi people of Ogale-Eleme; It is an area where almost all the water that flows during rainfall settles”. This is also demonstrated in agricultural produce harvested in impacted communities. Participants in the household survey stated that the growth characteristics of plants in their communities is an indication of overall agricultural output. Participants (42%) noted that the plants/trees were mostly stunted with black or no leaves, while others reported that the plants had slightly stunted shoots with black and yellow leaves (Table 5).

**Table 5:** Participants description of plants in their communities

Nature of plants/tree	Frequency	Percentages
Mostly tall green shoots	22	8.18
Medium sized shoot and green and yellowish leaves	55	20.45
Slightly stunted shoot with black and yellow leaves	104	38.66
Mostly stunted with black or no leaves	112	41.64

A participant at an FGD asserted: “The impact has been so much great that three plots of land cannot produce 10 basins of garri, we work harder with low yield”. Agricultural yield is reportedly low, while the size of produce such as cassava and yam is characteristically thin. The cocoyam, a staple crop in high demand locally called “togosugu”, was said to be locally extinct due to the effect of oil contamination. The plant is not grown or cultivated anymore in Ogoni.

It is difficult to practice shifting cultivation due to oil pollution and climate change on available land. The level of pollution has affected farming system, plant growth, and crop yield. Shifting cultivation was a common practice among farmers in Ogoni before the advent of incessant oil pollution in the area. Farmers in the area conventionally cultivate their farmland and after harvest allow the farmland to lie fallow for a period of 3 to 5 years. During the period, the farmer cultivates other arable lands. With extensive oil pollution on farmlands, farmers no longer practice shifting cultivation given the limited availability of clean land.

### Impacts of contamination on wildlife

Participants at the FGDs and household surveys indicated that wildlife was more abundant and closer to their communities prior to oil spills in Ogoniland. Animals such as lions, antelopes, grasscutters, elephants, monkeys, bush pigs, alligators, and crocodiles were abundant. Fish, birds, and other animals that were adversely affected by oil spills include oysters, bloody cockles (*bivalves*), mudskippers, periwinkles, vultures, eaglets (*awala*), porcupines (*bina*), rabbits, foxes (*gbe*), and grasscutters (*deelu*). Due to changes in vegetation cover, some fast-moving animals had migrated to “a more favourable” ecosystem. Participants reported declining catches of prawns, crayfishes, mullets, mudskippers, and crabs in their creeks. “The dry season used to be a major fish harvest season, but now the case is different, characterised by very low catches; oil patches are left on the shores”, commented one of the participants. According to an elderly male participant, ‘‘we used to hear certain bird sounds, but now we no longer hear them’’ implying that such birds had gone extinct locally or migrated to more favourable areas and regions.

Another participant, human rights watch dog, recounted that “apart from the disappearance of some species, there is a marked decline in the population of charismatic species around the neighborhood, such as snakes and water turtles”. The fisherfolk reported smelling petroleum in some fin and shell fishes caught in their creeks. Similar observations had earlier been reported in the Ogoniland (AI and CEHRD 2011; CEHRD 2019).

The changes in the environment, particularly habitat alteration, disturbance, or degradation, impact the local species’ community structure and create favourable habitat conditions for invasive species. The respondents posit that resilient wildlife such as monkeys, grasscutter, and squirrels that were commonly sighted are becoming increasingly scarce. A 65-year-old hunter exclaimed, ‘‘there is no more wildlife in our forest!’’. The biodiversity of Ogoniland is in dire need of conservation and restoration. UNEP (2011) noted that the mangrove wetlands of Ogoniland are potential Ramsar sites (wetland site of international importance), if the degraded environments are effectively restored and sustainable protection mechanisms are put in place. The UN Decade of Ecosystem Restoration presents an opportunity for restoring degraded habitats in Ogoniland.

### Impacts of oil contamination on livelihoods

**Fig. 9:**
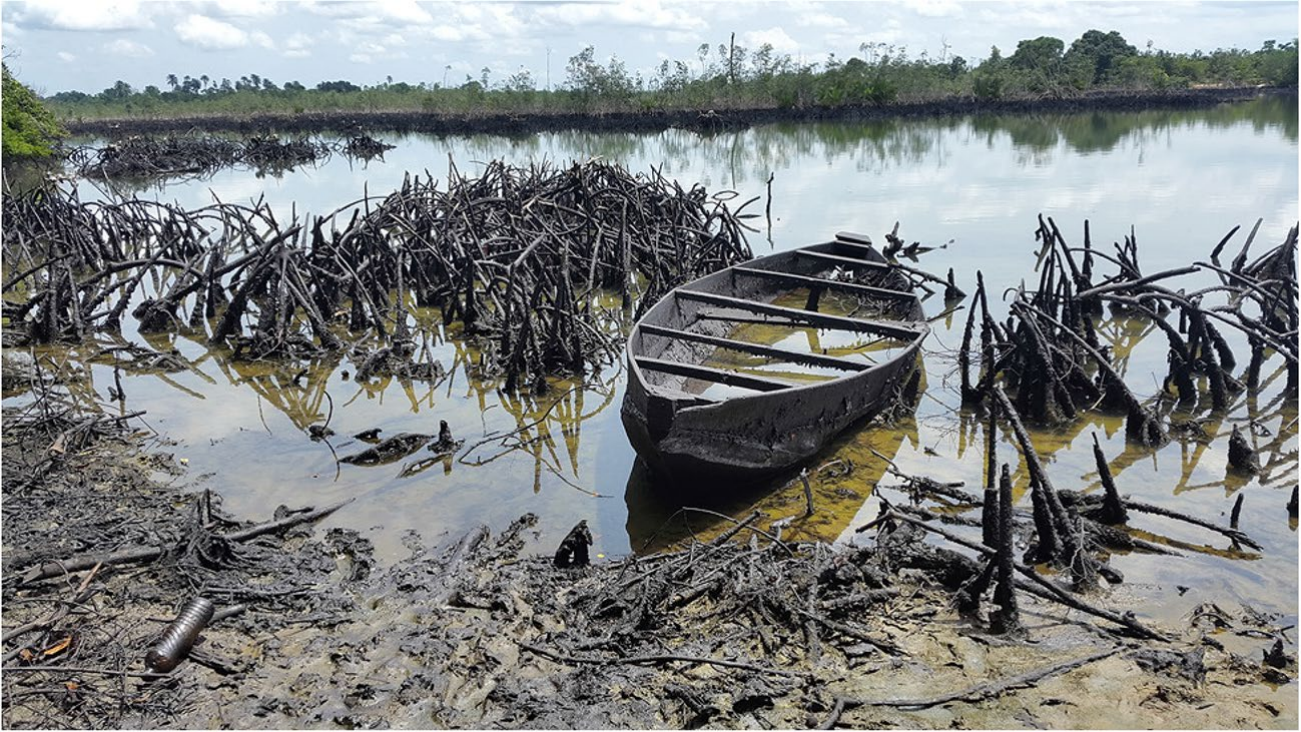
Destroyed livelihood (fish landing site) in Kporghor community

As indicated in Table 6, oil contamination has affected the main livelihood of the Ogoni people, which are farming and fishing. Most of the fishing is done in the rivers (49%), followed by creeks (38.29%), and streams (36,43%), while only 4% of fishing is done in the nearshore sea environment due to poor fishing gear and lack of fishers’ capacity to access the sea. Many of the fishing grounds have been destroyed by oil spills (see Fig. 9). About 91% of the respondents affirmed that their fishes smell and taste oil, thereby reducing the market value, and potentially pose health risk to consumers. Most respondents (89%) affirmed that oil contamination had changed their livelihood. People no longer catch subsistence fish in the contiguous creeks and rivers (Sam and Zabbey 2018). The option of fishing further afield in the lower reaches of the Bonny River and the Atlantic Ocean nearshore is limited by the requisite crafts and gear, as well as by the threats of a rough sea and piracy. Seventy-eight percent of the respondents explained that, given the reduced fish landings, they only fish occasionally, not necessarily for a livelihood as in the past. This has long-term implications for the age-old fishing culture of the people (Sam et al. 2017b). In Ogoniland, the indigenous fishing knowledge is transferred from one generation to another through social scaffolding, whereby the younger ones learn the act of fishing through subtle participation (Fentiman and Zabbey 2015). This includes taking a child along on fishing trips to bail out water from the canoe. The child then learns to fish by watching the more experienced older fisherwomen and fishermen. Fentiman and Zabbey (2015) reported that the inability to organize the annual “dor bon fish fencing party” in the Bodo Creek since 2008, when the creek was hit by two major oil spills, is eroding the fishing knowledge of the community and has created a knowledge gap that might be lost completely or take considerable time to recover.

**Table 6:** Impact of crude oil Pollution on livelihood in Ogoniland

Description of livelihood activities	Yes counts	%	No counts	%	Total no
Do you have family members that fish	209	77.70	60	22.30	269
Fishing is the main livelihood activity	114	42.38	155	57.62	269
Do the fish smell/taste oil	236	90.77	24	9.23	260
Farming the main/only livelihood	154	57.25	115	42.75	269
Oil contamination changed livelihood	241	89.59	28	10.41	269
Oil contamination affected farming	246	91.45	23	8.55	269
Contamination affected crop yield	258	99.23	2	0.77	260

**Fig. 10:**
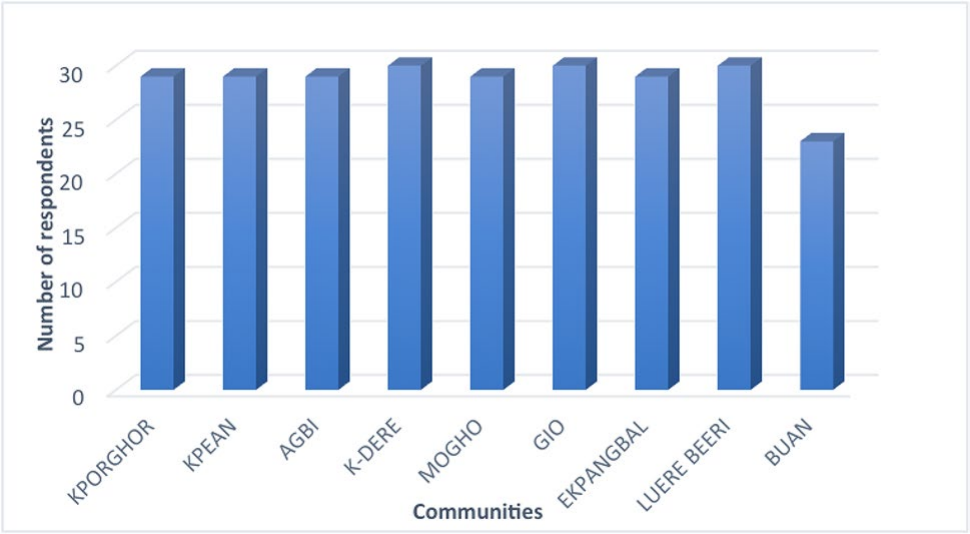
Effect of oil contamination on crop yield as indicated by respondents from different communities to the household survey

**Fig. 11:**
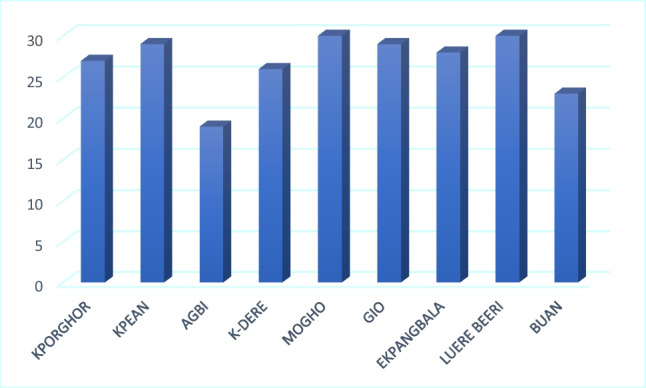
Effect of oil contamination on livelihood as indicated by respondents to the household survey

Similarly, 57.25% of respondents claimed that farming was their only source of livelihood. Oil contamination has affected farming, according to 91.45% of the household survey respondents. Contamination of arable land had crippled farming in Ogoniland and resulted in poor yields (Figs. 10 and 11). This was affirmed by 99% of the respondents from different communities, as shown in Table 6.

**Fig. 12:**
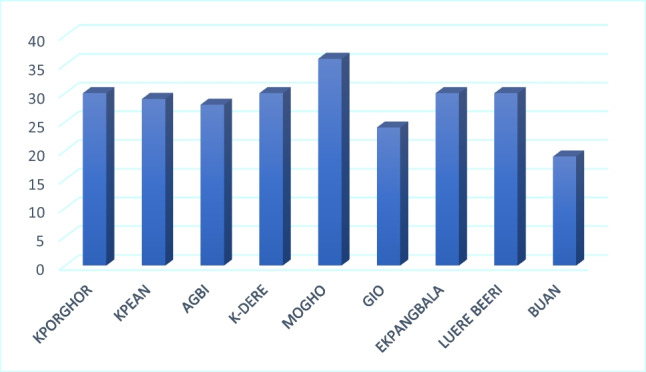
Oil contamination affect farming as indicated by respondents to the household survey

Nearly all 30 respondents from each community except Buan reported that oil contamination had affected their farming practices negatively (Fig. 12). This could be due to the sparse nature of contamination in Buan community. Also, the majority of oil infrastructures in the Buan community are distant from farmland, and as a result, oil spills often affect those in close proximity (Sam et al. 2017c).

Ejiba et al. (2016) argued that oil pollution in the Niger Delta had negatively impacted the population, causing damage to the livelihoods (mostly farming and fishing) in the communities. Oil pollution makes households more vulnerable and impact their wellbeing. Albert et al. (2018) indicated that the immediate environment suffers more than 90% environmental and socio-economic degradation. Furthermore, it is recognized that not every adaptation strategy works for every community. Adaptation strategy is therefore community specific, based on their livelihood strengths and options (Vincent–Akpu and Annor-Frempong 2017).

Similarly, Blythe et al. (2014) identified two livelihood strategies adopted by threatened coastal communities in Mozambique including intensification of fishing efforts and diversification. In Ogoni communities, they adapt to the oil pollution of their environment by intensifying their fishing by going farther into the sea. They also diversify their livelihoods and migrate to other cities for work due to urban pull factors.

### Livelihood Adaptations in Ogoni

To earn a living, respondents reported that they have developed alternative livelihoods for themselves. These alternative livelihoods include transportation businesses on motorbikes (locally called “okada riding”), trading, and artisanship. “Community members stated that they sometimes have to sell their land to buy the motor bike”. Some respondents commented that some of their community members have moved to urban centres to do menial jobs. The women’s group explained that their boys used to go fishing in the river, but they cannot find anymore. They go farther into the sea to fish, exposing them to the hazards of the high seas (Sam and Zabbey 2018). The women used to pick periwinkle and cut mangrove, but they can no longer do so because the creeks became polluted and the mangroves have degraded (Fig. 9). The women engage in tailoring, hairdressing, petty trading, and prostitution and serve as labourers in other people’s farms, while the youth are involved in mason, okada riding, and criminal activities. Members from Luere Beeri mainly engage in mat-making (from screw pine, *Pandunus candelabrum*), and this is one of their ancestral professions.

Improvised alternatives are not always sustainable and legitimate. Some community members are engaged in unsustainable sources of livelihood that are prone to crime. For example, several coastal communities are involved in artisanal refining, also known locally as “kpo fire”—a small-scale crude method of boiling hydrocarbons at different temperatures to derive kerosene, petrol, and diesel with significant waste release into the environment. This trade exacerbates the impacts of the already-contaminated environment. A security operative during the FGD commented thus “kpo-fire is a serious problem we have to address. Kpo-fire from what we know is able to contaminate areas that have been cleaned and we have a responsibility to ensure that doe not happen”. Concerns of re-pollution from the activities of artisanal crude oil refiners are an increasing problem that would determine whether or not HYPREP succeeds and thus forms a critical parameter to be monitored when evaluating HYPREP’s success (Sam et al. 2022).

The artisanal refining industry seems to have grown and attracted investors across stakeholder groups, and it is a potential factor that would affect a meaningful and sustainable clean-up exercise. Some communities are involved in the act and consider it a veritable alternative to a legitimate livelihood such as farming or fishing (Komi et al. 2019). For example, many communities have given social license to those involved in artisanal crude oil refining activities in efforts to engender alternative livelihood systems (Sam et al. 2022). Thus, as an aftermath of socio-economic impacts, increased artisanal refineries are one of the indicators that would indicate the success of remediation and restoration interventions in Ogoniland and the wider Niger Delta region. In addition, there is a high likelihood of re-polluting remediated land if the issue of artisanal refining is sufficiently addressed prior to clean-up activities. The problem of re-pollution from artisanal refining sites is a serious concern to all stakeholders within and outside the communities (Sam et al. 2022).

The proposal to issue licenses for modular refineries promised by the government of Nigeria could be a welcomed development for the people of Ogoni (Onuah et al. 2017). They considered the modular refinery as important as the clean-up process. A modular refinery is a prefabricated refinery constructed in modules and connected together by interstitial piping to form an easily manageable structure. This preference as well as the community involvement are symptoms of a feeling shared by communities that the oil wealth is not benefiting them, but rather impoverishing them while profiting others.

### Waste disposal

In general, communities lack designated dump sites for their waste, which is mostly made-up of domestic materials that they deposit on farms, while the non-biodegradable waste are burned or collected by waste pickers (scavengers). A government regulator during the FGD remarked that “we have a responsibility to ensure hazardous waste is controlled, our major concern is the limited awareness within the communities.” There is therefore a concern that hazardous waste generation will increase exponentially during the clean-up exercise and that such wastes will be disposed of in burrow pits or combined with domestic waste and disposed of in open dumps or farmlands.

The possibility also exists that contractors undertaking the clean-up might acquire or lease landed properties from landowners for the purpose of hazardous waste disposal. However, 92% of respondents in the household survey indicated that they have not been approached to lease their land properties. Increased waste generation is expected, and a specialized waste collection and management infrastructure constitute metrics to be considered as baselines for sustainable waste management systems.

### Towards a framework for monitoring a sustainable contaminated land management in the Niger Delta

Considering the various socio-economic parameters identified and discussed in this research, a conceptual outline of contextual parameters (Table 7) could support remediation practitioners and regulatory agencies to identify specific areas of interest that could enhance achieving stakeholders’ expectations. Table 7 therefore provides metrics for monitoring ongoing remediation projects in the Niger Delta region, specifically in Ogoniland. Most items outlined in Table 7 constitute areas of urgent intervention to alleviate sufferings and bring succour to oil-impacted communities. For example, the provision of alternative sustainable livelihoods and access to basic amenities such as potable drinking water are urgent priorities for these oil-impacted communities (Sam et al. 2024).

**Table 7:** Baseline socio-economic parameters to be monitored in the Ogoni remediation project

Parameter	Baseline	Expected change	Reference
Governance structure	Weak institutions with overlapping responsibilities	An independent agency with the requisite capacity to undertake remediation and restoration	UNEP 2011; Sam et al. (2015); Ambituuni et al. (2014)
Regulatory threshold	Target and intervention values set at 50 mg/kg and 5000 mg/kg, respectively	A downward review of the target value as recommended by UNEP. A ratification of bespoke threshold by the regulator	Sam et al. (2022)
Contaminated land management policy/regulation	Weak policy and outdated regulatory framework	A revamped regulation executed by an independent regulatory agency	Rim-Rukeh (2015); Ogunkan (2022)
Sustainable remediation parameters	There are no consensus parameters or frameworks for sustainable remediation in the Nigerian context	Development of a SuRF-Nigeria similar to SuRF-UK	Azuazu et al. (2023); Bardos et al. (2016)
Sustainable funding mechanism for contaminated land management	The Petroleum Industry Act highlights the initiation of an Environmental Remediation fund	This needs to be moved to the Ministry of Environment to be managed by NOSDRA	Sam et al. (2017a)
Potable drinking water	Water quality compromised by varying concentrations of hydrocarbon in surface and groundwater sources across communities in Ogoniland	Restoration of water quality that meet WHO and national water quality thresholds	UNEP (2011)
Soil and sediment quality	Soil and sediment quality compromised by elevated contaminant levels in soils at various locations across Ogoniland	Soil and sediment remediation to meet regulatory or bespoke thresholds ratified by the regulator	UNEP (2011)
Access to potable water for domestic use	Communities spend their monies to buy water they cannot attest the quality or source for domestic use	Access to free and potable water supply for domestic use by communities	Sam et al. (2022)
Livelihoods	A transition from farming and fishing to artisanal refining, motor bikes, artisanship and other social vices	Development of sustainable livelihood ventures that will encourage return to farming and fishing	Komi et al. (2019); Sam et al. (2024); Sam and Zabbey, (2018); Fentiman and Zabbey, (2015)
Mangrove ecosystem	Devastation of over 5000 hectares of mangroves	A restoration of mangrove habitats in degraded communities	Onyena and Sam (2020); Sam et al. (2023a); Numbere (2021)
Wildlife	Disappearance of wildlife in communities	A return of endemic wildlife species in the communities following sustainable ecosystem restoration	UNEP (2011)
Waste disposal	Indiscriminate dumping of mixed waste by road sides and in the rivers	Development of effective waste management systems that meets the twenty-first century requirements	Sagbara et al. (2020)
Re-pollution of remediated sites	Re-contamination or oiling of areas/shoreline that has been remediated	Limited cases of re-pollution as a result of alternative livelihoods provided to kpo-fire practitioners	Sam et al. (2024); Sam et al. (2022)

It should be noted that the intention of the current research is to not to provide a comprehensive metric for the monitoring and evaluation of remediation projects in the region but to highlight principal parameters that should be considered when evaluating ecosystem restoration in the Niger. For example, developing participatory platforms that would support inclusive deliberations towards reaching consensus on parameters that should constitute sustainable remediation in the Nigerian context similar to the Sustainable Remediation Forum (SuRF), UK, is highly needed (Azuazu et al. 2023). There is an increased understanding by decision-makers to remediate contaminated sites in the region, and thus, a SuRF-Nigeria will promote sustainable remediation of contaminated environmental media and support decision-makers to identify stakeholder expectations and potentially meet them.

## Conclusion and recommendations

With the confidence that the Nigerian Government intends to undertake a sustainable remediation and restoration intervention in Ogoniland and the Niger Delta region, it became imperative to provide a baseline or reference threshold for socio-economic parameters in Ogoniland. The study concludes that the socio-economic landscape of the Ogoni people, their livelihood, and the quality/standard of their lives have been compromised. Thus, there is a need for the immediate provision of potable drinking water, revision of the regulatory threshold, sustainable funding, restoration of mangrove ecosystem, elimination of re-pollution, and the establishment of an integrated water supply system as well as promotion of the provision of alternative livelihoods for all Ogoni communities. Livelihoods of local communities have been altered, and the environment has lost value for fishing, farming, and comfort. Any intervention in this area should take a comprehensive approach to developing and managing water supplies for the multiple uses and livelihood restoration. This is particularly important as government has begun survey for provision of potable drinking water to local communities in the area. Re-pollution is the single major challenge of the Ogoni clean-up; the government should therefore prioritize training and re-training of artisanal refiners in sustainable skills to reduce their impact on the clean-up process. Adequate waste management systems should be put in place in the communities because huge amounts of waste will be generated during the clean-up. It is expected that this study would be used as a reference material to measure the successes of the remediation and restoration processes in Ogoniland. Therefore, the study outlined initial baseline that would constitute parameters to be considered in the monitoring of the remediation process and indicative changes that would reflect success of the remediation and restoration project.

## Data Availability

Datasets collected and analysed during the current study have been used and could be shared without restriction. No additional data to be shared for the purpose of this publication.
